# Tuberculosis treatment and resulting abnormal blood glucose: a scoping review of studies from 1981 - 2021

**DOI:** 10.1080/16549716.2022.2114146

**Published:** 2022-09-30

**Authors:** Victor Williams, Chukwuemeka Onwuchekwa, Alinda G. Vos, Diederick E. Grobbee, Kennedy Otwombe, Kerstin Klipstein-Grobusch

**Affiliations:** a Julius Global Health, Julius Center for Health Sciences and Primary Care, University Medical Center Utrecht, Utrecht University, Utrecht, The Netherlands; b Monitoring and Evaluation Unit, National Tuberculosis Control Programme, Manzini, Eswatini; c Division of Epidemiology and Biostatistics, School of Public Health, Faculty of Health Sciences, University of the Witwatersrand, Johannesburg, South Africa; d International Health Department, Barcelona Institute of Global Health, Barcelona, Spain; e Ezintsha, Faculty of Health Sciences, University of the Witwatersrand, Johannesburg, South Africa; f Perinatal HIV Research Unit, Faculty of Health Sciences, University of the Witwatersrand, Johannesburg, South Africa

**Keywords:** Diabetes, hyperglycaemia, impaired glucose tolerance, human immunodeficiency virus

## Abstract

**Background:**

Hyperglycaemia is a risk factor for tuberculosis. Evidence of changes in blood glucose levels during and after tuberculosis treatment is unclear.

**Objective:**

To compile evidence of changes in blood glucose during and after tuberculosis treatment and the effects of elevated blood glucose changes on treatment outcomes in previously normoglycaemic patients.

**Methods:**

Original research studies (1980 to 2021) were identified in PubMed, Web of Science, CINAHL and Embase databases.

**Results:**

Of the 1,277 articles extracted, 14 were included in the final review. All the studies were observational and 50% were prospective. Fasting blood sugar was the most common clinical test (64%), followed by the glycated haemoglobin test and the oral glucose tolerance test (each 50%). Most tests were conducted at baseline and in the third month of treatment. Twelve studies showed that the prevalence of hyperglycaemia in previously normoglycaemic patients decreased from baseline to follow-up and end of treatment. Three studies showed successful treatment outcomes of 64%, 75% and 95%. Patients with hyperglycaemia at baseline were more likely to develop cavitary lung lesions and poor treatment outcomes and had higher post-treatment mortality. There was no difference in outcomes by human immunodeficiency virus (HIV) status.

**Conclusion:**

Elevated blood glucose in normoglycaemic patients receiving treatment for tuberculosis decreased by the end of treatment. Positive HIV status did not affect glucose changes during treatment. Further research is needed to investigate post-treatment morbidity in patients with baseline hyperglycaemia and the effects of HIV on the association between blood glucose and tuberculosis.

## Background

The World Health Organization estimates that ten million people were infected with tuberculosis (TB) in 2020, with 1.5 million deaths in the same year [1]. Concurrently, the International Diabetes Federation estimates that 537 million adults aged 20 to 79 were living with diabetes mellitus (DM) in 2021, with 75% of these residing in low- and middle-income countries (LMICs) [2,3]. An estimated 6.7 million people died from DM in 2021, with rising cases of type 2 diabetes mostly from LMICs [2,3]. Different studies indicate that people with DM are more likely to develop TB with worse treatment outcomes when receiving treatment for TB [4–7]. Therefore, understanding the blood sugar changes in patients during TB treatment is essential to ensure good treatment outcomes. Globally, the prevalence of TB among DM patients is estimated to be 15.3% [8]. This prevalence varies depending on the age and sex of the population, the burden of DM and TB in the population and human development index scores [8]. The prevalence of DM in active TB is highest in North America and the Caribbean (19.7%), Western Pacific (19.4%) and Southeast Asia (19.0%) compared with Africa (8.0%) [8]. A prevalence of 15%, 11% and 10% has been documented in Nigeria, Tanzania and Ethiopia, respectively [9].

While numerous studies indicate that diabetes is a risk factor for TB, it is not completely clear if TB or its treatment predisposes one to develop DM [10–12]. Available explanation points to an impaired glucose tolerance (IGT) during treatment with anti-TB drugs, which may or may not resolve once the treatment is completed [11,13–17]. This IGT is thought to be due to underlying undiagnosed diabetes or stress response from infection, resulting in increased levels of stress hormones, interleukin-1, interleukin-6 and TNF-alpha, abnormal functioning of the pancreas and possible TB-induced pancreatitis offsetting endocrine function [10,11,18]. Although plausible, these explanations have not been fully verified. Also, a high TB burden has been associated with human immunodeficiency virus (HIV) infection, which results in an immunocompromised state. So, HIV co-infection in TB patients may result in varied immune and endocrine responses with untoward outcomes.

Although studies describing the effect of TB treatment on blood glucose are available, these are few in Africa and other LMICs. Additionally, DM-TB studies in resource-poor settings with high HIV burden are required to understand the intersection with HIV. Some available studies have methodological limitations, such as small sample size and short follow-up post TB treatment [19–21] and were conducted before the HIV epidemic. A search of the PubMed and the Joanna Briggs Institute (JBI) Database of Systematic Reviews and Implementation Reports conducted on 25 July 2021 indicates few review articles and a systematic review protocol are available [10,18,22]. No scoping reviews were identified. The review articles presented useful information on the possible aetiology of abnormal glucose during TB treatment but none on the predictors. The available studies also focused on blood glucose changes in people with known DM status, not those with a normal blood glucose level before commencing TB treatment. Patients with known blood glucose anomalies will receive special care during TB treatment, but those presumed to have normal blood glucose may have poor treatment outcomes if abnormal changes in blood glucose were missed during treatment.

Therefore, the objective of this scoping review was to identify and compile the available evidence on possible abnormalities in blood glucose in previously normoglycaemic patients during and after TB treatment, using studies published from 1980 to 2021.

## Methods

This review was developed using the JBI reviewer’s manual and the methodology is based on the framework developed by Arksey and O’Malley [23,24].

### Scoping review questions

The following questions were used as a guide to fully describe the topic of this scoping review and the articles included in the review.

1. What methodology has been employed in describing the abnormal blood glucose arising from TB treatment?
2. What approaches have been identified as appropriate for measuring blood glucose during TB treatment?
3. What is the TB treatment outcome for patients who develop abnormal blood glucose while on TB treatment?
4. What factors determine the occurrence of abnormal blood glucose during TB treatment?
5. What is the frequency of abnormal glucose tolerance or DM in patients receiving TB treatment?

### Information sources and search strategy

A search was done for studies describing TB treatment’s effect on patients’ glucose levels from 1 January 1980 to 30 June 2021. This period was chosen to accommodate the increase in HIV infections that led to an increase in the number of new TB cases [25,26]. We searched the PubMed, Web of Science, CINAHL and Embase databases. A three-step approach was used to identify articles for inclusion in the review [23]. The first step was a preliminary search that involved identifying index terms and MeSH terms by searching PubMed and Embase using keywords from the scoping review’s topic (e.g. *tuberculosis treatment, TB treatment, abnormal glucose/hyperglycemia/glucose intolerance, diabetes*). The second step was to search the databases for articles using all the identified text words and keywords. The PubMed search was done on 8 November 2021 and the search terms used include (‘Tuberculosis’[MeSH Terms] OR ‘tuberculo*’[Title/Abstract]) AND (‘treat*’[Text Word] OR ‘therap*’[Text Word] OR ‘drug*’[Title/Abstract] OR ‘medication*’[Title/Abstract] OR ‘medicine’[Title/Abstract] OR ‘therapeutics’[MeSH Terms] OR ‘drug therapy’[MeSH Subheading]) AND (‘hyperglyc*’[Title/Abstract] OR ‘glucose intoler*’[Title/Abstract] OR ‘high blood glucose*’[Title/Abstract] OR ‘glucose tolerance’[Title/Abstract] OR ‘glycaemic’[Title/Abstract] OR ‘glycemic’[Title/Abstract] OR ‘hyperglycemia’[MeSH Terms] OR ‘blood glucose’[MeSH Terms] OR ‘Glucose Tolerance Test’[MeSH Terms]). Additional search criteria for other databases are available in ***Supplementary file 1***. The third step involved searching the reference list of the identified articles from the second step for additional articles for inclusion in the list of potential articles. Where there was a need, the authors of primary studies were contacted to obtain additional information regarding their study. A librarian from Utrecht University Library guided the search processes to ensure we used appropriate search terms and obtained relevant articles.

### Inclusion criteria

Included articles were original studies (case–control studies, cross-sectional studies, cohort studies and clinical trials) with participants of all ages from any part of the world. The following additional inclusion criteria were applied: (a) studies that were published from 1 January 1980 to 30 June 2021, (b) articles in English, (c) studies that specifically indicated that blood glucose was done at baseline or before the start of TB treatment and non-diabetic patients were followed-up either during or after treatment or both, and (d) studies that had information on the country where the study was conducted or specifically stated the region covered.

### Exclusion criteria

Excluded articles were those that were outside the study period, non-original studies (case reports, review papers, modelling studies, systematic reviews and meta-analyses, letters to the editor and opinion papers), studies for which the full texts were not accessible, studies with participants already known to be on treatment for DM, studies with no follow-up data and those with outcomes other than TB.

### Study selection for inclusion

**Figure 1.**
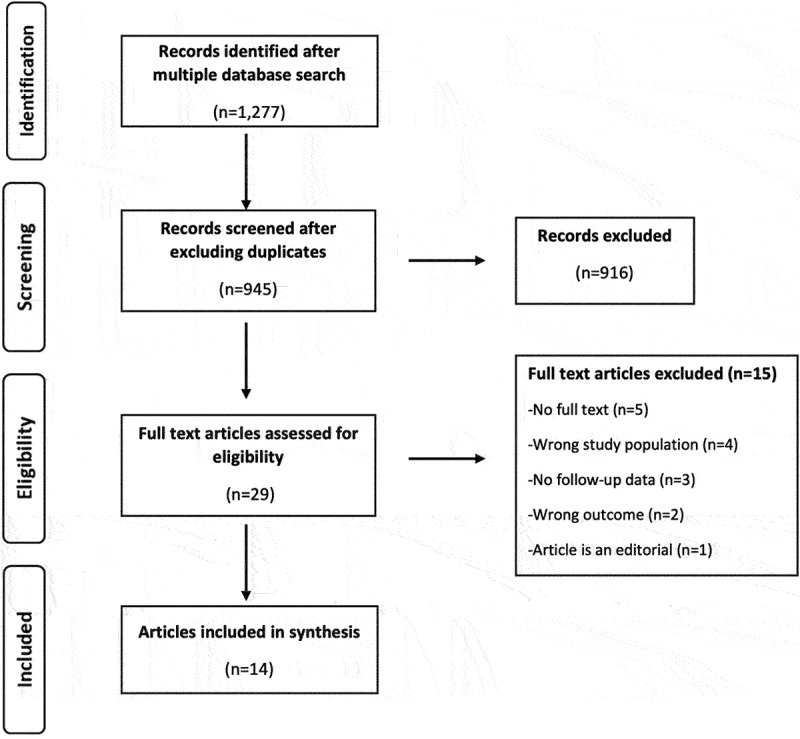
PRISMA flow diagram.

The study selection followed two steps. The first step was the title and abstract screening, and the second step was the full-text screening. All identified articles were compiled and entered into EndNote (Clarivate Analytics, Philadelphia, USA) for deduplication. Once the deduplication was complete, the remaining articles were uploaded into Rayyan software for title and abstract screening based on the inclusion criteria [27]. This was done independently by two reviewers (VW, CO). Where there was a conflict and the two reviewers could not agree, a third reviewer (AV) resolved the conflict. The full text of all the articles selected at the title/abstract stage was compiled and entered into EndNote for a full-text review and selection based on the inclusion criteria independently by two reviewers. Articles not meeting the inclusion criteria were excluded at this stage. The two reviewers first discussed and resolved disagreements and only invited the third reviewer when they did not agree. A PRISMA flow diagram (Figure 1) describes the steps adopted during article screening and selection for inclusion in the final study.

### Data extraction (Charting the results)

The information extracted from each article is listed in Box 1 and is based on the JBI reviewer’s manual [23]. A standardised data extraction form to capture the required information was developed in REDCap as a survey [28] (***Supplementary file 2***). This was validated and updated by two reviewers (VW, CO) using five selected studies per JBI guidance [23]. They independently extracted data from each article into the REDCap survey (each reviewer assigned each study a predetermined code to enable comparison). At the end of data extraction, data from the REDCap spreadsheet were compared, and all discrepancies were resolved before using a merged file for data synthesis and subsequent analysis. For clarity, a successful TB treatment outcome was defined as ‘cured or completed treatment’, while a poor outcome was defined as ‘relapse/treatment failure, loss to follow-up or death’.

Box 1.

1. Author(s)
2. Year of publication
3. Location the study was conducted (country, continent)
4. Aim/purpose
5. Study population (including mean/median age and sex)
6. Sample size
7. Study type (observational/experimental)
8. Study design used (including methods & time of glucose measurement)
9. Statistical method (descriptive/simple analysis/advanced)
10. Outcome details of the study (proportion with DM and hyperglycaemia, HIV status)

## Results

We identified 1,277 titles from our search (Figure 1). Of these, 945 unique titles were identified for screening after excluding duplicates. In the title and abstract screening, 916 articles did not meet the inclusion criteria, leaving 29 articles for a full-text review. Fourteen articles [13–15,19–21,29–36] were included in the final selection, while 15 articles were excluded. Two of the five authors with contact information whose main text was unavailable were contacted but did not respond. Contact information was not available for the other three.

### Description of included studies

The general characteristics of the 14 included studies are summarised in Table 1. Studies were conducted between 1984 and 2020, mainly in Asian (50%) and African (36%) countries. One study was conducted in South America and Europe. The articles, though varied, all aimed at studying or identifying IGT or hyperglycaemia during TB treatment. The studies were all observational, and 50% (n = 7) were prospective cohort studies. Twenty-one percent (n = 3) were case–control studies, and 14% (n = 2) were a combination of cross-sectional and prospective cohort studies. The sample size for the studies varied from 21 to 6,312, and participants from ten studies were patients receiving treatment for drug-sensitive TB. Of the remaining four studies, each used either multidrug-resistant TB patients (MDR-TB), HIV-TB co-infected patients, patients attending a private clinic or patients with respiratory symptoms. The participants were mostly males, with the proportion of males ranging from 49% to 78%, and the mean age of all participants ranged from 29.5 to 53 years.

**Table 1.** Description of the included studies.

FIRST AUTHOR (YEAR/COUNTRY)	AIM OF THE STUDY	STUDY DESIGN	SAMPLE SIZE	STUDY POPULATION	SEX (% MALES)	SIMPLE ANALYSIS*	ADVANCED ANALYSIS**
Purohit, S D (1984/India)	To assess the effect of rifampicin therapy on glucose tolerance	Prospective cohort	57	TB patients	77	Yes	No
Singh, M.M (1984/India)	To determine the prevalence of impaired glucose tolerance in active pulmonary TB patients and to determine the effect of anti-tuberculous chemotherapy on the glucose tolerance curves	Prospective cohort	52	TB patients	65	Yes	No
Oluboyo, P.O (1990/Nigeria)	To determine the significance of glucose intolerance in TB	Case-control study	54	Pulmonary TB patients	63	Yes	No
Jawad, F (1995/Pakistan)	To unmask glucose intolerance in patients with active pulmonary TB and to assess the effect of treatment on its reversal	Cross-sectional and prospective cohort	106	Outpatient pulmonary TB patients	59	No	No
Basoglu, O.K (1999/Turkey)	To compare glucose tolerance test results of pulmonary TB patients with those of patients with community-acquired pneumonia	Case-control study	58	Pulmonary TB patients	78	Yes	No
Tarbasi, P (2014/Iran)	To examine HbA1c of new TB patients and relate to if they complete TB treatment	Prospective cohort	158	New TB patients	49	Yes	No
Akinlade, K.S (2016/Nigeria)	To assess changes in glycated haemoglobin levels in MDR-TB patients	Longitudinal study	21	MDR-TB patients	68	Yes	No
Boillat-Blanco, N (2016/Tanzania)	To examine the association of TB and its outcome with the presence and persistence of hyperglycaemia in Tanzania, using three different DM screening tests.	Case-control study	530	Pulmonary TB patients	58	Yes	Yes
Lin, Y (2017/China)	To understand if blood glucose levels were stable or fluctuated during TB treatment	Prospective cohort	270	TB patients	66	Yes	Yes
Moreira, J (2018/Brazil)	To assess the impact/prevalence of hyperglycaemia on TB outcomes, comparison of treatment outcomes & one-year mortality rate based on the glycaemic status of patients during treatment	Retrospective cohort	473	HIV-TB coinfected patients	69	Yes	Yes
Diarra, B^#^ (2019/Mali)	To determine the prevalence of DM in newly diagnosed TB patients	Cross-sectional and prospective cohort	201	TB patients	73	Yes	Yes
Krishnappa, D (2019/India)	To determine the presence of hyperglycaemia (DM & IGT) in TB patients and assess outcomes after successful treatment	Prospective longitudinal study	582	TB patients	56	Yes	No
Habib, S.S (2020/Pakistan)	To investigate the outcome of bidirectional TB-DM screening in the private sector	Cross-sectional	6312	All patients attending a private clinic	53	No	Yes
Kubjane, M (2020/South Africa)	To assess the association between hyperglycaemia and TB, at TB diagnosis, and after three months of TB treatment	Prospective cohort	850	Patients with respiratory symptoms	53	Yes	Yes

^#^Patients without DM at baseline were followed-up at months two and five.

*Chi-square, t-test, ANOVA, Fisher’s Exact, correlation, Mann–Whitney test, Kruskal–Wallis test

**Linear/Logistic/Multilinear regressions/Cox regression

Seven out of the 14 studies (50%) included HIV-co-infected participants. The proportion of HIV co-infection in the four studies was less than 10%, then 26%, 61% and 100% in the remaining three studies. In ten studies, participants received first-line TB treatment, one was a second-line only and three were all types of treatment.

### Method of glucose estimation

Four main types of glucose estimation tests were used either singly or in combination. These include FBS (64%), glycated haemoglobin test (HbA1c) (50%), oral glucose tolerance test (OGTT) (50%) and random blood sugar test (RBS) (14%). Some studies combined two or more tests to estimate glucose levels: 36% (FBS + OGTT), 21% (FBS + HbA1c), 14% (HbA1c + OGTT), 14% (RBS + HbA1c), 7% (FBS + HbA1c + OGTT) and 7% (RBS + HbA1c + OGTT).

### Time of glucose estimation

**Figure 2.**
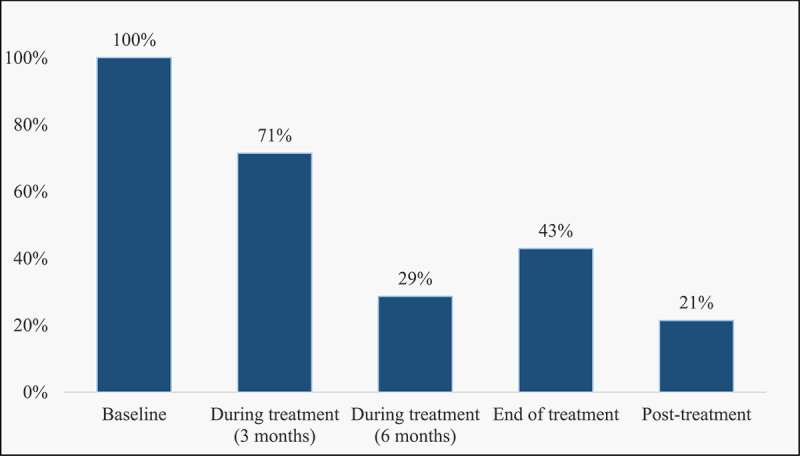
Timelines* for glucose estimation in different studies.

Five parameters described the time of glucose estimation in the studies: baseline, three months, six months, end of treatment and post-treatment (Figure 2, Table 2). Measurements were done at baseline in all 14 studies and a combination of time points thereafter. Two studies (14%) used all five parameters to describe the time of glucose estimation.

**Table 2.** Glucose changes before and during treatment.

*FIRST AUTHOR(YEAR)*	*SAMPLE SIZE*	*TIME OF GLUCOSE ESTIMATION*	*BASELINE DM (%)*	*BASELINE* *IGT (%)*	*PARTICIPANTS AT FOLLOW-UP (N)*	*% DM AT FOLLOW-UP*	*% IGT AT FOLLOW-UP*
Purohit, S D (1984)	57	0, 7, 30 days	-	10.0	0	0.0	0.0
Singh, M.M (1984)	52	0, 4, 8, 12 weeks	3.8	44.2	52	1.9	11.5
Oluboyo, P.O (1990)	54	0, 3, 3 months PT^#^	5.6	37.0	53	1.9	9.3
Jawad, F (1995)	106	0, ET	19.8	29.2	23	21.7	21.7
Basoglu, O.K (1999)	58	0, 3 months	8.6	10.3	58	0	0
Tarbasi, P (2014)	158	0, 3 months	0	31.0	158	24.0	34
Akinlade, K.S*(2016)	21	0, 2, 4, 6 months PT	-	-	-	-	
Boillat-Blanco, N (2016) **	530	0, ET	6.8	24.3	378	1.4	10
Lin, Y (2017)	232	0, 2, 6 months	0	7.3	232	0	3.0
Moreira, J (2018)	473	0, 3, 6 months, ET, PT	2.1	10.4	426	5.4	-
Diarra, B (2019)	201	0, 2, 5 months	5.5	3.0	190	0	-
Krishnappa, D (2019)	582	0, ET, PT	7.0	4	579	-	1.5
Habib, S.S (2020)	6312	0, 3 months	24	32	502	-	42.0
Kubjane, M (2020)	850	0, 3 months	11.9	46.9	276	9.3	21.5

*Two months follow-up showed a significant reduction in mean HbA1c. No values were indicated.

**Reported values are for 2-hCG (two-hour capillary glucose). **^#^**In those with abnormal results. **ET** – End of treatment; **PT** – Post-treatment.

Follow-up glucose at 30 days and two months was grouped with three months

*Will not sum up to 100% since studies used a combination of timelines.

### Glucose changes during tuberculosis treatment

Most of the studies defined DM and hyperglycaemia based on the guidance provided by the American Diabetes Association [37]. In this guide, DM is defined as glucose level ≥7.0 mmol/l, ≥11.1 mmol/l or ≥6.5% using FBS, OGTT or HbA1c, respectively. IGT is similarly defined as a glucose level of 5.6–6.9 mmol/l, 7.8–11.0 mmol/l or 5.7–6.4% using FBS, OGTT or HbA1c, respectively. The studies excluded patients with a known diagnosis of DM before conducting a baseline glucose test. With some variability, patients identified with glucose levels consistent with DM and hyperglycaemia had repeat tests at specified periods. Table 2 describes the proportion of participants with DM and hyperglycaemia at baseline and during the follow-up period.

Twelve (86%) studies showed the proportion of previously normoglycaemic patients with glucose values in the DM and IGT range at baseline reduced during treatment follow-up and end of treatment, while only two studies [15,35] showed an increase (Table 2). DM decreased from 11.9% at baseline to 9.3% at follow-up, while IGT decreased from 46.9% at baseline to 21.5% at follow-up [36] in one of the studies conducted in South Africa. On the contrary, an Iranian cohort study [15] showed that 24% of patients developed DM in the follow-up period, while the proportion with IGT increased from 31% to 34%. Similarly, another study in Pakistan [35] observed that the proportion of IGT increased from 32% at baseline to 42% at follow-up. Most of the follow-up was done at three months (71%) followed by end of treatment (43%). With follow-up at different times, most studies (86%) agree there is a reduction in the glucose level at follow-up compared to baseline and dysglycaemia observed at baseline normalised at follow-up or end of treatment. Glucose levels were higher in older patients, mostly above 40 years, compared to younger patients [13–15].

### TB treatment outcome and glucose changes

A summary of results with TB treatment outcomes and glucose changes is presented in Table 3. In three studies, 64%, 75% and 95% of the patients had a successful treatment outcome [15,33,34]. Two studies indicated that TB patients with DM or IGT were more likely to develop cavitary lung lesions, with one of the studies indicating a 54% prevalence [15,30]. In one study, where patients were followed up to one year after TB treatment, patients with hyperglycaemia had a 48.9% risk of mortality compared to 7.9% in those with euglycaemia [33]. While another study showed that hyperglycaemia at enrolment diagnosed using fasting capillary glucose was associated with poor treatment outcomes, such as loss to follow-up, treatment failure or death (aOR 2.46; 95% CI: 1.08 to 5.57) [21], a 2019 study from Mali [34] indicates that blood sugar levels had no impact on TB treatment outcomes. Researchers in Nigeria [31] did not find any difference in HbA1c levels based on HIV status, but a 2017 study in China [32] showed an HIV positive status, DM, smoking cigarettes and presenting to a hospital instead of a clinic were associated with an unstable FBS during TB treatment.

**Table 3.** Summary of results from the different studies.

FIRST AUTHOR (YEAR/COUNTRY)	OVERALL SUMMARY AND TREATMENT OUTCOME
Purohit, S.D (1984/India)	The mean rise in glucose was lower on the 30^th^ day compared to the pre-treatment rise. The mean rise in glucose was higher in the rifampicin group at different intervals compared to the baseline.
Singh, M.M (1984/India)	54% (19/35) of those with cavities had an impaired glucose tolerance indicating an association between cavities and IGT. Only six out of 23 with initial impaired glucose tolerance continued to show impairment at 12 weeks.
Oluboyo, P.O (1990/Nigeria)*	Only 3.7% of patients remained abnormal three months after full treatment compared to baseline. The result suggests glucose intolerance during TB treatment caused by infection and is reversible. All patients had improved symptomatic and radiographic features at the end of treatment.
Jawad, F (1995/Pakistan)*	Glucose levels improved and returned to normal after TB treatment.
Basoglu, O.K (1999/Turkey)*	OGTT results returned to normal in both TB and pneumonia groups after treatment. Cases with abnormal OGTT were older than 40 years and more likely to be males.
Tarbasi, P (2014/Iran)*	95% had a successful treatment outcome. 24% developed DM, were older, had the highest level of FBG and had the highest prevalence of cavitary lung lesions.
Akinlade, K.S (2016/Nigeria)	HbA1c decreased at two months post-treatment compared to baseline. There were no changes in glucose levels at months four and six of treatment compared to baseline. No difference was seen in HbA1c levels based on HIV status.
Boillat-Blanco, N (2016/Tanzania)	DM or IGT at enrolment was significantly associated with adverse TB outcomes (i.e. loss to follow-up, treatment failure, or death).
Lin, Y (2017/China)	HIV positive status, DM, smoking cigarettes and presenting at a hospital rather than a clinic increased the odds of association with unstable FBG.
Moreira, J (2018/Brazil)*	75% successful treatment outcome. Hyperglycaemia was associated with an increased risk of mortality one year after TB treatment compared to euglycaemia (48.9% vs 7.9%).
Diarra, B (2019/Mali)*	No elevated blood sugar was seen at follow-up for the two periods. 64% with DM had a good TB treatment outcome and blood sugar levels had no impact on treatment outcome.
Krishnappa, D (2019/India)	Patients with hyperglycaemia (DM & IGT) were older. The blood sugar levels improved in all patients with DM following treatment of TB.
Habib, S.S (2020/Pakistan)	42% (213/502) with previous normal HbA1c had an increased HbA1c at three months follow-up while 58% (141/244) with previous elevated HbA1c dropped to the normal range at three months follow-up.
Kubjane, M (2020/South Africa)	2.6% (n = 10) with DM at enrolment reverted to normal at follow-up; and 22.5% (n = 105) of patients with IGR reverted to normal at follow-up.

*Studies with outcomes

### Outcomes in TB-HIV co-infected patients

Of the seven studies that included HIV co-infected participants, six provided information on glucose changes or their association with TB treatment outcomes based on the HIV status of the participants. The different outcomes are presented in Table 4.

**Table 4.** Summary outcomes in HIV-positive patients receiving TB treatment.

FIRST AUTHOR(YEAR/COUNTRY)	STUDY POPULATION	SAMPLE SIZE	% HIV POSITIVE	OUTCOME BASED ON HIV STATUS
Tarbasi, P (2014/Iran)	New TB patients	158	3.2	Not assessed.
Akinlade, K.S (2016/Nigeria)	MDR-TB patients	21	26.3	No difference in baseline HbA1c between HIV-positive and negative participants (p = 0.954).
Boillat-Blanco, N (2016/Tanzania)	Pulmonary TB patients	530	32	Using HbA1c, HIV-positive participants had lower odds of DM (p = 0.048). No difference between those receiving ART and those not receiving ART.
Lin, Y (2017/China)	TB Patients	270	3.3	Only 9/151 with known HIV status were HIV positive and 6/9 HIV positive patients had unstable FBG. HIV-positive participants had higher odds of unstable FBG after adjusting for confounding (p = 0.027).
Moreira, J (2018/Brazil)	HIV-TB coinfected patients	473	100	***Successful treatment outcome*** – euglycaemic group (75%), hyperglycaemia group (28%), DM group (80%);***Adverse event*** – euglycaemic group (25%), hyperglycaemia group (71%), DM group (20%);***Death*** – euglycaemic group (7.5%), hyperglycaemia group (51%), DM group (10%);***Lost to follow-up*** – euglycaemic group (17%), hyperglycaemia group (20%), DM group (10%).
Diarra, B (2019/Mali)	TB patients	201	6.5	No HIV-positive participant developed DM during TB treatment.
Kubjane, M (2020/South Africa)	Patients with respiratory symptoms	850	61	Significant positive association between DM and TB at baseline (OR, 2.4 [95% CI, 1.3–4.3]) and follow-up (OR, 3.3 [95% CI, 1.5–7.3]) regardless of HIV status.

## Discussion

This scoping review has compiled findings from different studies on the changes in blood glucose levels of patients receiving treatment for TB. Most of the studies were conducted in Asia and Africa (Table 1), indicating locations with a high prevalence of TB. Consistent with the known epidemiology of TB, there were more male participants in the studies than females, and glucose levels were higher in older participants. The FBG test was the commonest method for estimating blood sugar, followed by OGTT and HbA1c. There was no standardised approach to estimating blood sugar for patients, and most studies combined two or more approaches. In the studies where a combination of tests was used, HbA1c had higher values and patients with baseline values in the DM or IGT range were more likely to persist as hyperglycaemia throughout treatment [21]. This further indicates the use of HbA1c in identifying patients with a long-term glucose abnormality.

Although all studies conducted baseline blood glucose assessments, subsequent measurements were different across the studies. For glucose screening to identify DM comorbidity during treatment, the timing of blood glucose screening should be standardised to allow for comparison across different patients and country programmes. Some studies only repeated glucose measurements for patients who were not known DM patients, but with glucose measurements in the DM or IGT range at baseline, excluding those with normal baseline values [20,21,35]. These studies could have primarily aimed at following up on patients with abnormal glucose measurements or adopted as a cost-saving measure. A limitation of this approach is that new cases of DM or hyperglycaemia during the follow-up period could be missed.

Findings from this review suggest the mean blood glucose levels in patients who were previously not known to have DM but with baseline values in the DM or IGT range decreased once they commenced treatment. The prevalence of elevated blood glucose also decreased during follow-up. This is consistent with earlier findings that the elevated blood glucose at diagnosis may be due to stress hormones’ response to the disease process [10,11,18]. However, the elevated blood glucose did not always resolve following treatment, as some studies reported patients with persistent hyperglycaemia after TB treatment (Table 2). This could be people with undiagnosed DM before getting infected with TB or those already with IGT who develop DM due to the extra insulin resistance triggered by infection. Two studies conducted in Iran and Pakistan indicated an increase in blood sugar measurements after treatment [15,35]. We are cautious of the interpretation of these studies as the number of patients screened at follow-up was lower than the baseline. This reduced number at follow-up during TB treatment highlights a common problem encountered by TB programs where patients are lost to follow-up or discontinue treatment due to various reasons, such as distance to the health facility, stigma, treatment fatigue, relocation or treatment costs. Another reason could be down referral of patients once they are stable on treatment from tertiary health facilities to lower-level facilities such as clinics.

The development of cavitary lung lesions indicates the severe abnormality in the immune response during TB infection and could be associated with hyperglycaemia [30,38]. Two studies reported poor treatment outcomes (relapse, death or loss to follow-up) in patients with DM or hyperglycaemia at enrolment and one-year post-treatment follow-up [21,33]. This is consistent with a 2019 systematic review that showed the odds of death (OR 1.88, 95% CI 1.59–2.21) and relapse (OR 1.64, 95% CI 1.29–2.08) were higher in patients with DM receiving TB treatment compared to normoglycaemic TB patients [39]. Similarly, a 2022 multi-centre prospective cohort study from Brazil showed that poor TB treatment outcomes were associated with baseline dysglycaemia and higher HbA1c values [40]. From the studies, it is seen that glucose values improved over time with good TB treatment outcomes. A 2021 study from Ghana shows that though more patients with normoglycaemia had a sputum conversion at two months compared to those with hyperglycaemia, this difference became insignificant at six months, indicating that the observed dysglycaemia at the onset of treatment was temporary [41] and had no association with treatment outcomes. This implies that good treatment outcomes can often be achieved in DM patients with adequate glucose control.

This review assessed studies that included HIV-positive participants to ascertain if HIV status affected DM-TB association, but the findings were mixed, tending toward a reduction in hyperglycaemia or no difference based on HIV status (Table 4). This could be because we had only six studies reporting this, and it was not the primary outcome of our study. Despite this, conflicting findings have been reported on the effect of HIV on TB/DM or hyperglycaemia. Studies conducted in Tanzania and Nigeria [42–44] indicate a stronger association among HIV-negative participants, while another study conducted in South Africa [45] indicates a stronger association among people living with HIV. Further research is required to convincingly describe this association as the different blood glucose measurement approaches and medications taken by people living with HIV can influence outcomes [36].

### Strengths and limitations

A key strength of this scoping review is the rigorous methodological approach adopted at the different stages to ensure reproducibility, minimal errors and that the included studies met the inclusion criteria. The review team accessed four electronic databases to ensure relevant studies were not excluded. We also expanded the search to cover a period when HIV cases gradually increased and, more recently, to cover the period of the COVID-19 pandemic where we expect more screening for diabetes would be done since it is a high-risk factor for COVID-19-associated mortality. Finally, our review team have diverse expertise (infectious disease epidemiologists, biostatisticians, clinicians, public health specialists and non-communicable disease epidemiologists), which served as a useful resource to guide the review process.

As a limitation, our review included relatively few articles as most studies in this field assessed glucose changes in known DM patients receiving treatment for TB. Since we did not extract records from all the databases, we may have missed some studies from the databases we did not search. For studies published during the COVID-19 pandemic, bias may likely have been introduced by elevated dysglycaemia from COVID-19 infections. But these are few and published in the early days of the pandemic. No risk of bias assessment was done to ascertain the methodological rigour of the included studies; therefore, recommendations cannot be provided based on the findings of this review. Nevertheless, we have been able to present findings from studies that describe glucose changes in non-DM patients receiving treatment for TB.

## Conclusion

This scoping review aimed to identify and compile the available evidence on possible abnormalities in blood glucose during and after TB treatment. The studies indicated that dysglycaemia in patients receiving treatment for TB normalised after commencing anti-TB medication at the end of treatment, and a positive HIV status was not associated with glucose changes during TB treatment. There was no standardised method and time for testing or screening as the reviewed studies adopted different approaches. Further investigations on patient follow-up after TB treatment for possible signs of glucose changes that may result in high mortality and the impact of HIV on the association between DM and TB are required. This will enable definitive conclusions on the observed high mortality in persons with high glucose post-treatment and any effect of HIV on the association between DM and TB.

## Supplementary Material

Supplemental Material

Supplemental Material

## Paper context

Evidence of changes in blood glucose during tuberculosis treatment and the association with HIV is unclear. Our study shows that dysglycaemia identified at the onset of tuberculosis treatment is normalised at follow-up and end of treatment and patients with baseline dysglycaemia have poor outcomes post-treatment compared to normoglycaemic patients. HIV status was not associated with glucose changes during treatment. Further research is required to understand morbidity post-tuberculosis treatment and the association with HIV.
