# Supplementary material for: Tuberculosis treatment and resulting abnormal blood glucose: a scoping review of studies from 1981 - 2021
Source: Glob Health Action. 2022 Sep 30;15(1):2114146. doi: 10.1080/16549716.2022.2114146 (PMC9543146; doi:10.1080/16549716.2022.2114146)
Supplement: Supplemental Material [file ZGHA_A_2114146_SM6000.pdf]

Confidential

Scoping Review Data Extraction Form

Serial Number ______________________

Study Description

Assigned study code ______________________

Year of Publication ______________________

First Author (Surname & Initials) ______________________

Continent study was conducted
- ◯ Africa
- ◯ Antarctica
- ◯ Asia
- ◯ Australasia
- ◯ Europe
- ◯ North America
- ◯ South America

Country study was conducted ______________________

Overall aim of the study ______________________

Description of Study Participants

Specific study population ______________________

Type of TB Disease
- ◯ DSTB
- ◯ RRTB
- ◯ DRTB
- ◯ XDR
- ◯ All

(Single select)

Type of TB treatment
- ◯ First Line
- ◯ Second Line
- ◯ Third Line
- ◯ All

(Single select)

Proportion of HIV positive participants (%) ______________________
(Number Only)

Age (Mean/SD) ______________________

Age (Median/IQR) ______________________

% Female ______________________

% male ______________________

Study Design/ Methods

Study type
- ○ Observational
- ○ Experimental

Study design ______________________

Study /sample size ______________________

Sample size at follow up ______________________

Statistical analysis method
- ☐ Descriptive
- ☐ Simple analysis (Chi-square, t-test, ANOVA, correlation etc)
- ☐ Advanced (Linear/Logistic/Multi linear regressions etc)

(Multiselect)

Consideration (reasons) for selected analysis method ______________________

Glucose measurements and Timelines

Glucose level estimation method
- ☐ Urine dispstick
- ☐ FBS
- ☐ RBS
- ☐ Hba1c
- ☐ OGTT

(Multiselect)

Time of glucose estimation
- ☐ Baseline
- ☐ During treatment (3 Months)
- ☐ During treatment (6 Months)
- ☐ End of treatment
- ☐ Post treatment (During follow-up)

(Multiselect)

Proportion of participants with DM at baseline (%) ______________________

Proportion of participants with DM at follow-up (%) ______________________

Proportion of participants with impaired glucose tolerance (IGT) at baseline (%) ______________________

Proportion of participants with impaired glucose tolerance (IGT) at follow-up (%) __________________________________

Study Outcomes

Main outcome (summary) __________________________________

Relevant additional findings __________________________________

Outcome based on HIV status __________________________________

Additional comments on the study __________________________________
