# Supplementary material for: Tuberculosis treatment and resulting abnormal blood glucose: a scoping review of studies from 1981 - 2021
Source: Glob Health Action. 2022 Sep 30;15(1):2114146. doi: 10.1080/16549716.2022.2114146 (PMC9543146; doi:10.1080/16549716.2022.2114146)
Supplement: Supplemental Material [file ZGHA_A_2114146_SM5998.docx]

# Supplementary File 1: Additional Search Criteria for other databases

1. Web of SCIENCE

Date 8 November 8, 2021. Output: 570

ALL=(tuberculos*)

((((ALL=(treat*)) OR ALL=(drug therap*)) OR ALL=(therap*)) OR ALL=(medica*)) OR ALL=(medicine)

((((((((ALL=(hyperglyc*)) OR ALL=(glucose intoler*)) OR ALL=(high blood glucose*)) OR ALL=(glucose tolerance)) OR ALL=(glycaemi*)) OR ALL=(glycemi*)) OR ALL=(Hyperglycemia)) OR ALL=(Blood Glucose)) OR ALL=(Glucose Tolerance Test)

//////////////////////////////////////////////////////////////////////////////////////////////////////////////////////////////////

1. CINAHL

Date 8 November 8, 2021. Output:

TI tuberculos*

TI treat* OR TI drug therap* OR TI therap* OR TI medica* OR TI medicine

TI hyperglyc* OR TI glucose intoler* OR TI high blood glucose* OR TI glucose tolerance OR TI glycaemi* OR TI blood glucose OR TI glucose tolerance test

//////////////////////////////////////////////////////////////////////////////////////////////////////////////////////////////////////////////////////////////////

1. **Embase**

Date 9 November, 2021. Output: 356

1: 'tuberculosis'/exp OR tuberculo*

2: 'therapy'/exp OR therap*:ti,ab OR drug*:ti,ab OR medica*:ti,ab OR medicin*:ti,ab

3: hyperglyc*:ti,ab OR 'glucose intol*':ti,ab OR 'high blood glucose':ti,ab OR 'glucose tolerance':ti,ab OR glycemic:ti,ab OR glycaemic:ti,ab OR 'glucose tolerance test':ti,ab

1 AND 2 AND 3
